# Kaposi's sarcoma in mainland Tanzania: a report of 117 cases.

**DOI:** 10.1038/bjc.1969.45

**Published:** 1969-06

**Authors:** G. Slavin, H. M. Cameron, H. Singh

## Abstract

**Images:**


### KAPOSI'S SARCOMA IN MAINLAND TANZANIA: A REPORT OF 117 CASES

G. SLAVIN, H. McD. CAMERON AND H. SINGH

*From the University Department of Pathology, Glasgow Royal Infirmary, and the Muhimbili Hospital, Dar es Salaam, Tanzania*

Received for publication March 6, 1969

IN 1872, Moritz Kaposi described 5 patients with a multiple, pigmented sarcoma of skin and noted at autopsy, similar lesions in the gastro-intestinal tract, larynx and trachea (Kaposi, 1872). The disease was said to occur with greater frequency in patients from Eastern Europe and certain parts of Northern Italy (Rothman, 1962; de Amicis, 1882), and some reports indicate that it is more common in Jews (Rothman, 1962). It occurs rarely in Great Britain, most parts of Western Europe and the United States (Bluefarb, 1957; Oettlé, 1962). It has been said to be rare in Negroes (Bluefarb, 1957) and, whilst this may be correct in the United States, Kaposi's sarcoma occurs with great frequency in indigenous African Negroes (Thijs, 1957; Murray and Loethe, 1962*a*; McLean, 1963; Edington, 1956; Johnstone, 1965; Lee 1968). The frequency of the disease reported from various African centres is very varied (Fig. 1), and Quénum (1957) has

FIG. 1.—Incidence of Kaposi's sarcoma in histologically proven malignancies (Edington, 1956; Elmes and Baldwin, 1944; Thijs, 1957; Higginson and Oettlé, 1960; Timms, 1961; Lothe, 1963).

suggested that the condition increases in frequency as one approaches the Equator. Davies (1959) has stressed its infrequency in dry sandy areas and greater frequency in moist tropical areas.

### MATERIAL

This paper records our experience with Kaposi's sarcoma occurring in mainland Tanzania during the period January 1964 to June 1966. All the cases were diagnosed histologically at the Central Pathology Laboratory, Dar es Salaam. 117 cases are recorded. Clinical information was available from the biopsy request forms and this has been supplemented by further information obtained by writing to the hospitals from which the biopsies were sent. The Central Pathology Laboratory is the only histological laboratory centre in mainland Tanzania and receives biopsies from almost all hospitals, both Government and Mission. Zanzibar and Pemba have their own pathology service and do not figure in this report. All the biopsies are from mainland Tanzanian Africans. 2 cases came to autopsy.

*Frequency.* Kaposi's sarcoma occurs frequently in Tanzania and in 1964–65 accounted for 4% of all malignancies diagnosed by biopsy. It presents most commonly as a cutaneous lesion, and in our biopsy material is surpassed in frequency as a cutaneous malignancy only by squamous carcinoma and malignant melanoma.

*Sex.* 108 cases occurred in males and 9 in females.

*Age.* The age distributions of these cases on first admission to hospital is shown in Fig. 2. The disease occurs at all ages but most in the 4th to 7th decades. 8 cases in this series occurred in children less than 16 years of age. The presentation and course in children is discussed in detail elsewhere (Slavin *et al.*, 1969).

FIG. 2.—Age of patients on first admission to hospital.

*Clinical Presentation and Course*

The most common presentation (105 cases) was with cutaneous lesions. They occurred as raised warty nodular lesions of the skin most marked in peripheral distribution (Fig. 3) and occasionally noted to be distributed along the course of superficial veins. The lesions were usually small, 0·2–1 cm. in diameter, lying in the dermis or subcutis and bulging the overlaying epidermis (Fig. 4). Closely contiguous lesions sometimes became confluent and produced large conglomerate lesions. More diffuse lesions gave rise to plaque-like nodules. Whilst the lesions

FIG. 3.—Sites of cutaneous lesions of Kaposi's sarcoma. Note the well marked peripheral distribution of the lesions.

were principally in the subcutis or dermis, superficial ulceration was a noteworthy feature. A peculiar rather firm peripheral oedema was commonly noted in conjunction with the skin lesions (Fig. 5).

In 12 cases the initial presentation was enlargement of lymph nodes other than those regional to cutaneous lesions. Seven of these cases were in children (Fig. 6). Three were adults in whom solitary enlargement of a group of nodes was noted without skin lesions, and in 2 adults node lesions were associated with visceral lesions. Both these latter patients died and are discussed later.

Kaposi's sarcoma frequently runs a protracted course and death may follow from inter-current causes. Although we have little data on the course in this series some estimate of its progression is given by considering the duration of the disease preceding biopsy (Table I). Many lesions are present for years before biopsy, the longest being for 15 years. No relationship was noted between the clinical extent of the disease and the duration.

TABLE I.—*Duration of Disease Before Biopsy in 71 Cases in Which This Information was Available*

| Time (in years) | < 1 | 1 | 2 | 3 | 4 | 5 | 6 | 7 | 8 | 9 | 10 | 11 | 12 | 13 | 14 | 15 | . | "long duration" |
|---|---|---|---|---|---|---|---|---|---|---|---|---|---|---|---|---|---|---|
| Number of cases: | 23 | 16 | 12 | 6 | 1 | 2 | 2 | — | 2 | — | 1 | — | 1 | — | — | 1 | . | 4 |

Four deaths occurred during the period of study and these illustrate the possible fulminant course of the disease:

*Case I*

A male of 18 years presented with marked pitting oedema of both lower limbs associated with hepato-splenomegaly, cervical, inguinal, and axillary lymphadenopathy. His condition rapidly progressed and he died within 4 months of the onset. Biopsy of an inguinal node showed total destruction of the normal architecture, replaced in part by Hodgkin's disease while in adjacent fields separate and distinct typical Kaposi's tissue was noted. Permission for autopsy was not obtained in this case.

*Case II*

A previously healthy male aged 60 years was admitted to hospital as an acute abdominal emergency with intestinal obstruction. At laparotomy, the terminal ileum was thickened and obstructed for about 15 cm. of its length. The thickening extended into the mesentery which had a nodular surface. Numerous large retroperitoneal nodes had a haemorrhagic colour. No skin lesions were noted. Postoperatively he developed intractable congestive cardiac failure and died. Autopsy was not permitted.

Histological examination of the bowel showed Kaposi's sarcoma principally in the submucosa but extending through the wall and into the mesentery.

*Case III*

A male child of $2\frac{1}{2}$ years developed generalised lymph node enlargement and was diagnosed as Kaposi's sarcoma by biopsy. Despite treatment with nitrogen mustard he died within 6 months of the onset of his disease. Autopsy showed massive involvement of many lymph node systems, and visceral lesions in caecum, appendix and ileum.

EXPLANATION OF PLATES

FIG. 4.—Multiple nodules are seen bulging and ulcerating the skin. A large conglomerate lesion is seen on the right heel.

FIG. 5.—Oedema associated with sparse plaque like nodules on dorsum of the hand.

FIG. 6.—Gross enlargement of cervical, post-auricular and occipital lymph nodes by Kaposi's sarcoma in a child of $2\frac{1}{2}$ years.

FIG. 7.—Broad interweaving bundles of spindle cells with vascular clefts. H. and E. ×105.

FIG. 8.—Blood cells in vascular clefts are in direct contact with spindle cells, without intervening endothelium. H. and E. ×424.

FIG. 9.—Ectatic lymph spaces and inflammatory infiltrate at margin of lesion. H. and E. ×105.

FIG. 10.—Dermal nodule of Kaposi's sarcoma with characteristic separation from overlying epidermis by zone of normal tissue. H. and E. ×41.

4

5

6

Slavin, Cameron and Singh.

7

8

Slavin, Cameron and Singh.

9

10

*Case IV*

A male child of 10 years was admitted to hospital with a generalised lymphadenopathy of 11 months and several skin lesions on the abdomen, scrotum and penis. Diagnosis was made by biopsy. Despite treatment with nitrogen mustard he progressively deteriorated and died within 14 months of the onset of his disease. Autopsy showed massive involvement of the lymphatic system with grossly enlarged nodes and deposits in the spleen, heart, left adrenal and epiglottis.

*Cases III and IV* are reported in greater detail elsewhere (Slavin *et al.*, 1969).

*Associated diseases*

In case I, the concurrence of Kaposi's sarcoma and Hodgkin's sarcoma of a lymph node was seen. One other malignancy was noted in association with lesions of Kaposi's sarcoma: A woman had her right leg amputated because of histologically verified malignant change in a tropical ulcer. Enlarged inguinal nodes removed surgically were largely replaced by tissue typical of Kaposi's sarcoma.

*Histology*

Our material shows a characteristic pattern consisting of spindle cells set in broad interweaving bundles (Fig. 7). Between the cells vascular clefts without endothelial lining are seen and red blood cells are directly in contact with the tumour cells (Fig. 8). In other areas angiomatoid tissue both cavernous and capillary may be seen and particularly at the periphery large nutrient vessels may be noted. Dilated lymphatics are prominent at the edges of the lesion (Fig. 9). In several lesions avascular spindle cell areas are noted with a close appearance to fibrosarcoma. However, in such cases further biopsy has always revealed more typical areas. Inflammatory cells, mainly lymphocytes and plasma cells, are seen and this infiltration is most marked at the periphery where perivascular cuffing with plasma cells may be prominent. In some cases a sparse interstitial round cell infiltrate extends between the spindle cells.

At the edges the nodules tend to be well defined and are sometimes bounded and lobulated by adjacent fibrosis. In some cases the tumour is much more ill defined merging into the surrounding tissue. In the skin, early lesions lie in the middle and lower dermis with a band of unaffected dermis between the tumour and overlying epidermis (Fig. 10). In older lesions the tumours abut directly on to the epidermis, producing ulceration or inducing epithelial hyperplastic changes.

In the other organs examined in this series the histological features are identical with those in the skin. No difference is noted between the histological features of cases pursuing a fulminant course and those progressing more indolently.

### DISCUSSION

Kaposi's sarcoma occurs frequently in indigenous Africans. It is rare in the American Negro, and in non-Africans living in Africa. Oettlé (1962) has stressed that this immunity may be retained even when the immigrant population has been present in the community for over 3 centuries, as in South Africa. Of his 66 cases from Johannesburg, 63 were Bantu and only 3 were white people; none were Coloureds or Indians, and he mentions that the latter are subject to the same types of housing conditions, are found in similar townships, and attend the same hospitals. Nevertheless, he believes that genetic differences are relatively unimportant and emphasises the importance of environment; in particular, a common diet, infections and possibly occupational factors associated with race. In Tanzania it is a common malignancy, and in a study of the patterns of distribution of malignant neoplasms a greater frequency of the disease in the extreme North-west near to Rwanda, in the area immediately south of Lake Victoria and in the areas of the Southern Highlands has been described (Burkitt and Slavin, 1968). The reason for these local variations in frequency is not clear. It may be as McLean (1963) suggests that any such apparent variation in incidence of the disease reflects only differences in the available medical services in different areas but these variations may be significant and are worth study. In Uganda, Williams and Williams (1966), have described a greater incidence of Kaposi's sarcoma in those areas where infestation with onchocerca is heavy, and they speculate that Kaposi's sarcoma may be an infective condition spread by a vector.

In our material there is a marked preponderance of cutaneous lesions but as Kaposi described, it is a systemic disease. Visceral lesions noted by us are recorded in Table II but these are certainly under-estimated, because this is

TABLE II.—*Extracutaneous Sites of Kaposi's Sarcoma Seen in* 117 *Cases*

| | | | | |
|---|---|---|---|---|
| Lip | 1 | . | Spleen | 1 |
| Tongue | 1 | . | Adrenal | 1 |
| Uvula | 1 | . | Heart | 1 |
| Epiglottis | 1 | . | Lymph Nodes | 12 |
| Ileum | 2 | . | Bone | 1 |
| Caecum | 1 | . | Conjunctivae | 1 |
| Appendix | 1 | | | |

chiefly a biopsy series. In the only large autopsy series reported from Africa, Murray and Lothe (1962*b*) described lesions in all the viscera except the brain. In a biopsy series there is inevitable distortion of the true distribution of the disease in favour of skin and readily accessible biopsy sites.

In 12 cases lymph node involvement was a major or the only presenting complaint. 7 cases occurred in young children. Lymph node involvement has been considered uncommon (Ecklund and Valaitis, 1962), but the attention drawn to this presentation in Africans by Burke-Gaffney (1928) and Elmes (1954) has been emphasised by Davies and Lothe (1962) and Dutz and Stout (1960) who stress the relative frequency of lymph node lesions in young children.

The male : female ratio of 12 : 1 agrees closely with that described in other series. Lothe (1963) in Uganda, found only 2·9% of female cases and took pains to show this was not due to selection or local prejudice against the treatment of women in hospital. In children the male : female ratio is only 3 : 1. It is tempting to seek constitutional factors as the cause of these disparities. However, treatment of the disease with sex hormones does not support the view that there is any direct endocrine influence. Though Cook (1966) cites evidence that in an African setting such incidence can result from environmental causes, Hutt (1969, personal communication) after visiting areas characterised by a high incidence of Kaposi's sarcoma thinks that the observed sex differences are impossible to explain by any simple environmental factors.

The clinical course of Kaposi's sarcoma is often protracted as exemplified by many of our cases. However, it is unpredictable and may follow a rapidly fulminant and downhill course. In 4 cases death rapidly followed the onset of the disease. In 2 of these cases young children presented with massive lymphadenopathy and relatively minor cutaneous lesions. This course has been emphasised in African children by Davies and Lothe (1962) and Dutz and Stout (1960).

Spontaneous regression of the disease occurs uncommonly. It occurred only partially in one of our cases where the sclerosis and disappearance of old lesions were associated with the appearance of fresh lesions, more proximally on the limb.

Previous reports have emphasised the frequency of second primary neoplasms in association with Kaposi's sarcoma (Moertal and Hagedorn, 1957; Bluefarb, 1957). O'Brien and Brassfield (1966) followed 63 Caucasian patients with Kaposi's sarcoma and found that 18 died from the effects of a second primary neoplasm, including 5 with Hodgkin's disease, 3 with a lymphosarcoma, 1 with a malignant melanoma, 1 with myeloma and 8 with various carcinomas. This large series reveals an excessive incidence of second malignancies. However, they are of varied type and this series does not support the claim (Oettlé, 1962; Pack and Davis, 1954) that second primaries associated with Kaposi's sarcoma are predominantly reticulo-endothelial. In Africa second primary neoplasms have been recorded much less commonly. Lothe (1963) saw only 3 cases in his series of 291 cases of Kaposi's sarcoma. In 19 autopsy cases, Murray and Lothe (1962) described a bronchial carcinoma, a hepatoma and a case of Hodgkin's disease in association with Kaposi's sarcoma. Uys and Bennett (1959) have described a single African patient with coincident Hodgkin's disease and McKinney (1967) reports the coincidence of Kaposi's sarcoma and African lymphoma in a child. Thijs (1957) records the occurrence of lymphatic leukaemia and Kaposi's sarcoma in a male child of 4 years. In our material 2 associated second primary neoplasms are noted: a squamous carcinoma of leg, and Hodgkin's disease.

The nature of the spindle cells which form the tumour is obscure. Histochemical, electron microscopic, and tissue culture studies have failed to identify the cell of origin. Many results are contradictory, but most workers agree that the cells of the main layers of blood vessel walls are excluded as sources (Dorfman, 1962), and interest now centres on the adventitial coat, claims being made for the Schwann cell, modified nerves of the glomus body (Becker, 1962), and reticulo-endothelial cells (Dayan and Lewis, 1967). Davies (1962) thinks that opinion has swung towards the reticulo-endothelial system, but this is largely based on the alleged frequency of lymphomata in association with Kaposi's sarcoma. As already noted, where a second primary tumour is found, it is as likely to be epithelial as reticulo-endothelial in origin.

While most workers accept it as a true tumour of multicentric origin, some have reservations (Roulet, 1962) and Willis (1962) prefers to call the condition "Kaposi's Disease". Three features are of note in setting it apart from other neoplasms: (1) The well documented occurrence of spontaneous regression (MacKee and Ciporallo, 1936; Lothe, 1963). This may lend support to the suggestion that immune factors are involved in the pathogenesis of the disease (*Lancet*, 1967). Only one such case was encountered in this series. It should be noted, however, that regression is apparently never complete but, as happened in our example, as individual lesions disappear others form to take their place. (2) The dramatic male predominance, which is as yet unexplained. This does not appear to be due to selection of cases and there is no direct evidence for a hormonal factor. The male predominance is much less marked in children. (3) The occurrence of a second form of presentation, mostly in young patients, in whom the brunt of the disease is borne by lymph nodes rather than by skin. Again the significance of this is not known and it may be that an environmental study of such cases would yield clues as to the pathogenesis of the disease.

### SUMMARY

Kaposi's sarcoma accounts for 4% of malignancies diagnosed by biopsy in Tanzania. The clinico-pathological features of the disease in 117 African patients are presented and are discussed.

Our thanks are due to Dr. B. Akim, Chief Medical Officer, Ministry of Health, Tanzania, for permission to publish this material. We are grateful to Dr. J. Hammerton, Consultant Anaesthetist, Dar es Salaam, and Mr. T. Parker, Glasgow Royal Infirmary, for photographic assistance.

### REFERENCES

DE AMICIS, T.—(1882) Quoted by Ronchese (1958).
BECKER, J. F. P.—(1962) *Acta Un. int. Cancer.*, **18**, 164.
BLUEFARB, S. M.—(1957) 'Kaposi's Sarcoma'. Springfield, Illinois (Charles C. Thomas).
BURKE-GAFFNEY, H. J.—(1928) *A. med. sanit. Rep., Tanganyika.*
BURKITT, D. AND SLAVIN G.—(1968) in 'Cancer in Africa'. Nairobi (East African Publishing House).
COOK, J.—(1966) *Jl R. Coll. Surg. Edinb.*, **11**, 3.
DAVIES, J. N. P.—(1959) 'Modern Trends in Pathology', 1st edition. London (Butterworths).—(1962) *Acta Un. int. Cancr.*, **18**, 59.
DAVIES, J. N. P. AND LOTHE, F.—(1962) *Acta Un. int. Cancr.*, **18**, 81.
DAYAN, A. D. AND LEWIS, P. D. (1967) *Nature, Lond.*, **213**, 889.
DORFMAN, R. F.—(1962) *Acta Un. int. Cancr.*, **18**, 161.
DUTZ, W. AND STOUT, A. P.—(1960) *Cancer, N.Y.*, **13**, 1964.
ECKLUND, R. E. AND VALAITIS, J.—(1962) *Archs Path.*, **74**, 60.
EDINGTON, G. M.—(1956) *Br. J. Cancer*, **10**, 595.
ELMES, B. G. T.—(1954) *J. Path. Bact.*, **67**, 610.
ELMES, B. G. T. AND BALDWIN, R. B. T.—(1944) *Ann. trop. Med. Parasit.*, **41**, 321.
HIGGINSON, J. AND OETTLÉ, A. G.—(1960) *J. natn. Cancer. Inst.*, **24**, 589.
JOHNSTONE, G.—(1965) *J. trop. Med. Hyg.*, **68**, 1.
KAPOSI, M.—(1872) Quoted by Rothman (1962).
*Lancet*, Leading Article (1967) ii, 1290.
LEE, F. D.—(1968) *J. clin. Path.*, **21**, 119.
LOTHE, F.—(1963) 'Kaposi's sarcoma in Ugandan Africans'. Oslo (Universitetsforlaget).
McLEAN, U.—(1963) *Br. J. Cancer*, **17**, 195.
McKINNEY, B.—(1957) *E. Afr. med. J.*, **42**, 3.
McKEE, G. M. AND CIPORALLO, A. C.—(1936) *Am. J. Cancer*, **26**, 1.
MOERTAL, C. G. AND HAGEDORN, A. B.—(1957) *Blood*, **12**, 788.
MURRAY, J. F. AND LOTHE, F.—(1962*a*) *Acta Un. int. Cancr.*, **18**, 100.—(1962*b*) *Acta Un. int. Cancr.*, **18**, 116.
O'BRIEN, P. H. AND BRASSFIELD, R. D.—(1966) *Cancer, N.Y.*, **19**, 1497.
OETTLÉ, A. G.—(1962) *Acta Un. int. Cancr.*, **18**, 17.
PACK, G. T. AND DAVIS, J.—(1954) *Archs Derm. Syph.*, **69**, 604.

QUÉNUM, A.—(1957) ' La Maladie de Kaposie en Afrique Noire ' Bordeaux. Quoted by McLean (1963).
RONCHESE, F.—(1958) *A.M.A. Archs Derm.*, **79**, 336.
ROTHMAN, S.—(1962) *Acta Un. int. Cancr.*, **18**, 13.
ROULET, F.—(1962) *Acta Un. int. Cancr.*, **18**, 147.
SLAVIN, G., CAMERON, H. M., FORBES, C. AND MORTON MITCHELL, R.—(1969) (in press).
THIJS, A.—(1957) *Annls Soc. belge Méd. trop.*, **37**, 295.
TIMMS, G. L.—(1961) Quoted by Oettlé (1962).
UYS, C. J. AND BENNET, M. B.—(1959) *S. Afr. J. med. Sci.*, **5**, 39.
WILLIAMS, E. H. AND WILLIAMS, P. H.—(1966) *E. Afr. med. J.*, **43**, 6.
WILLIS, R. A.—(1962) ' The Pathology of Tumours of Children '. Edinburgh (Oliver and Boyd).


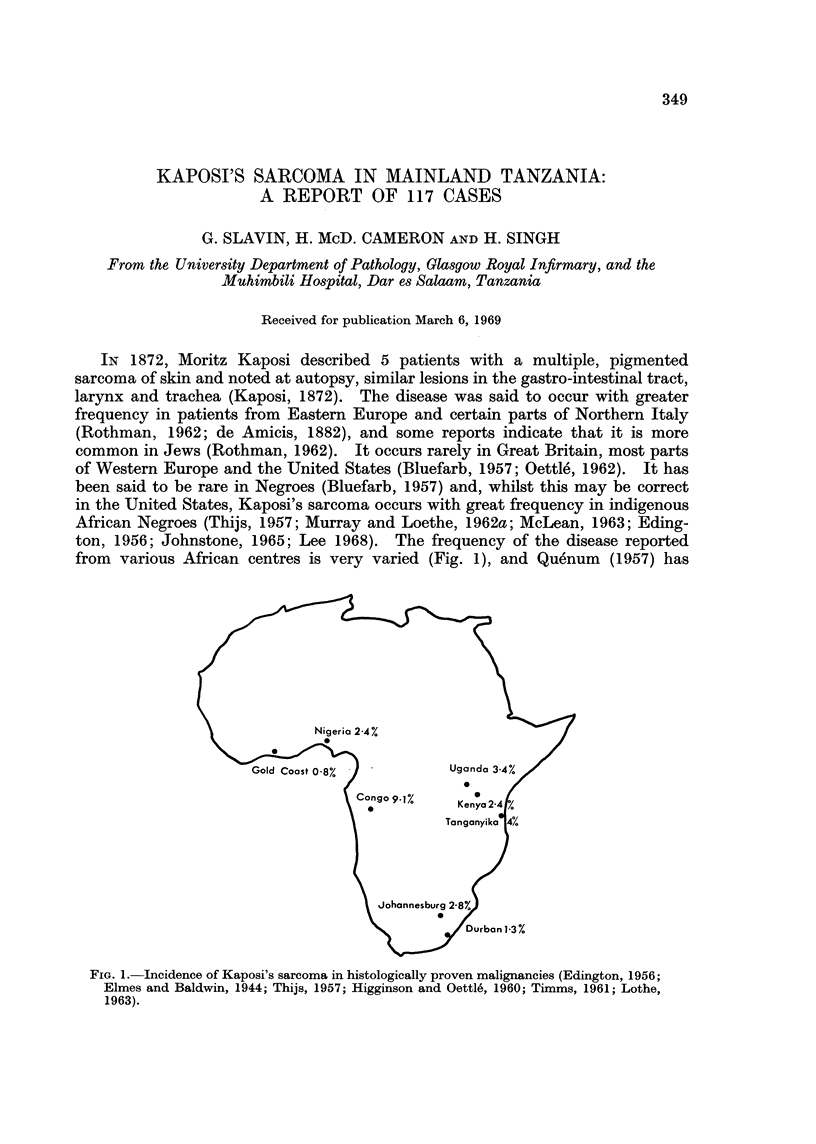

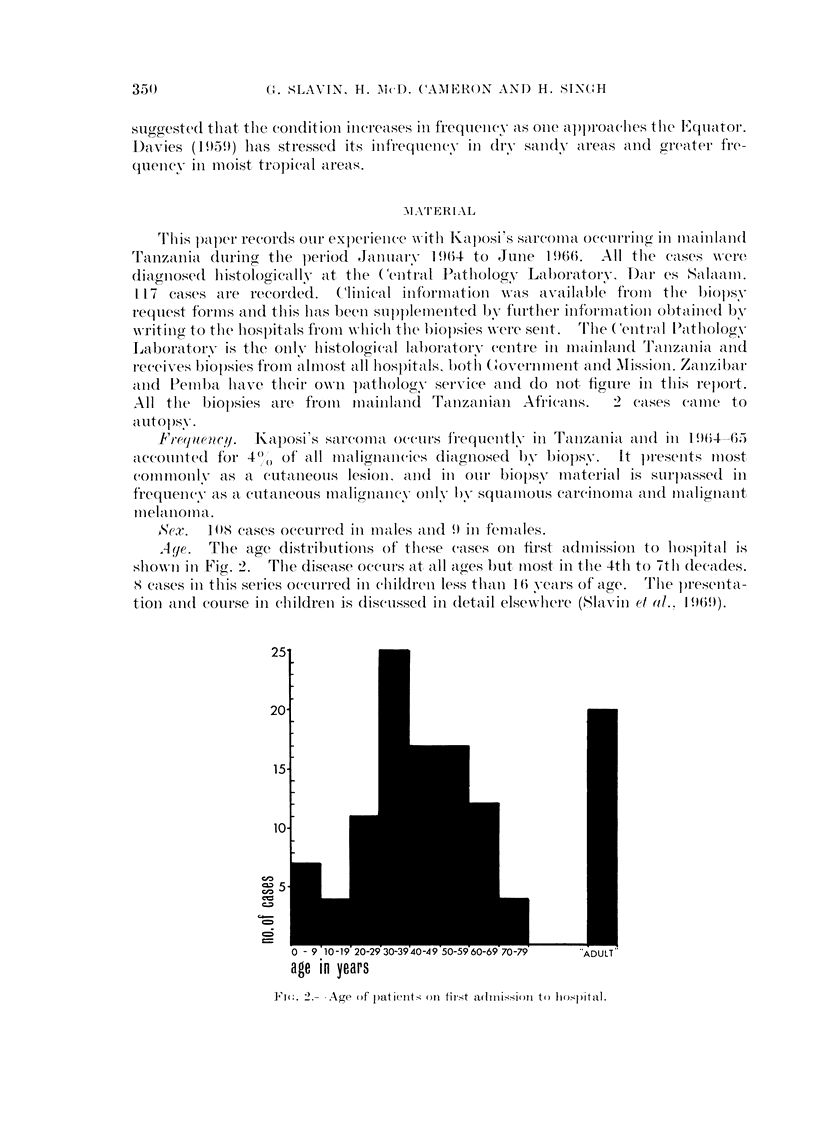

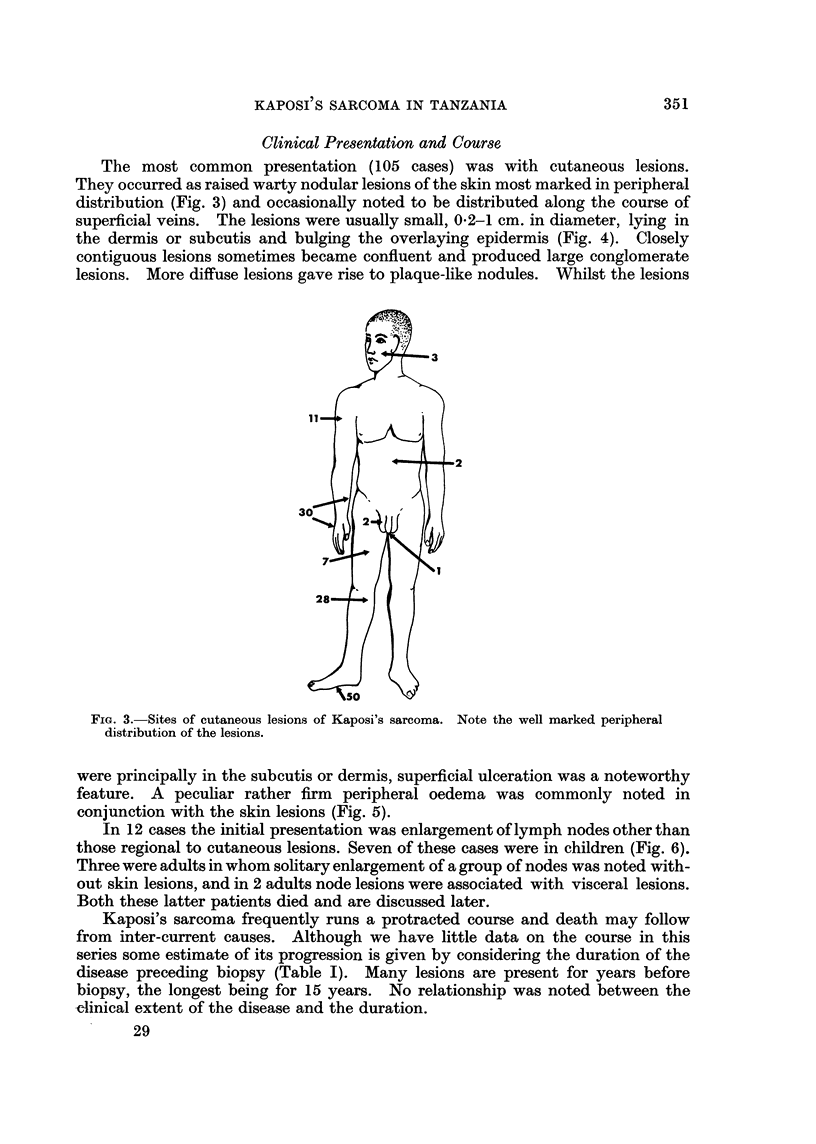

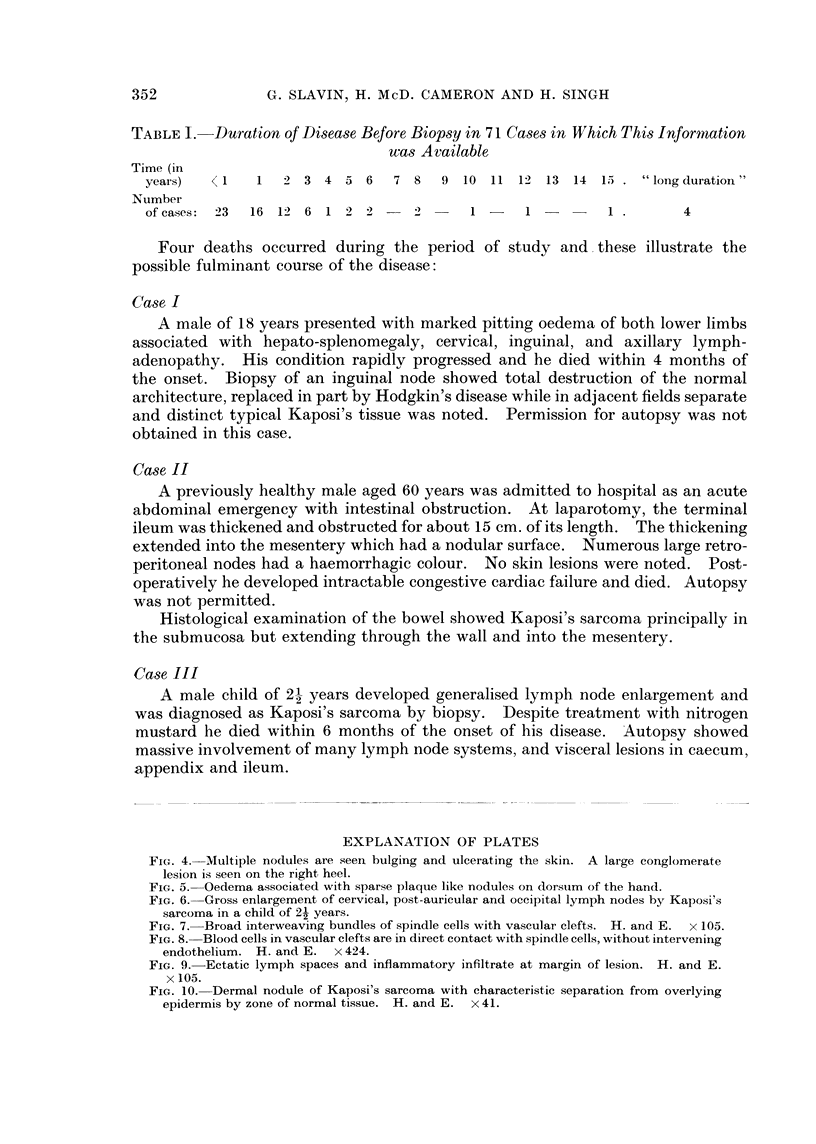

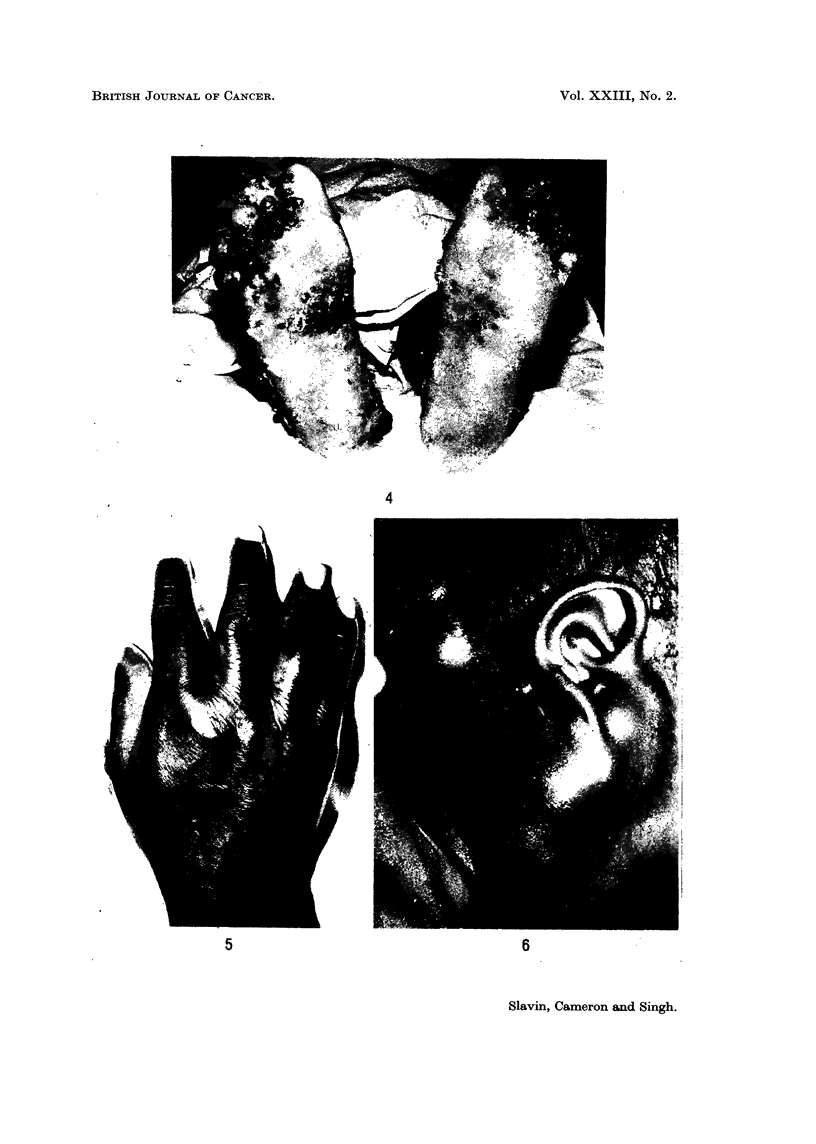

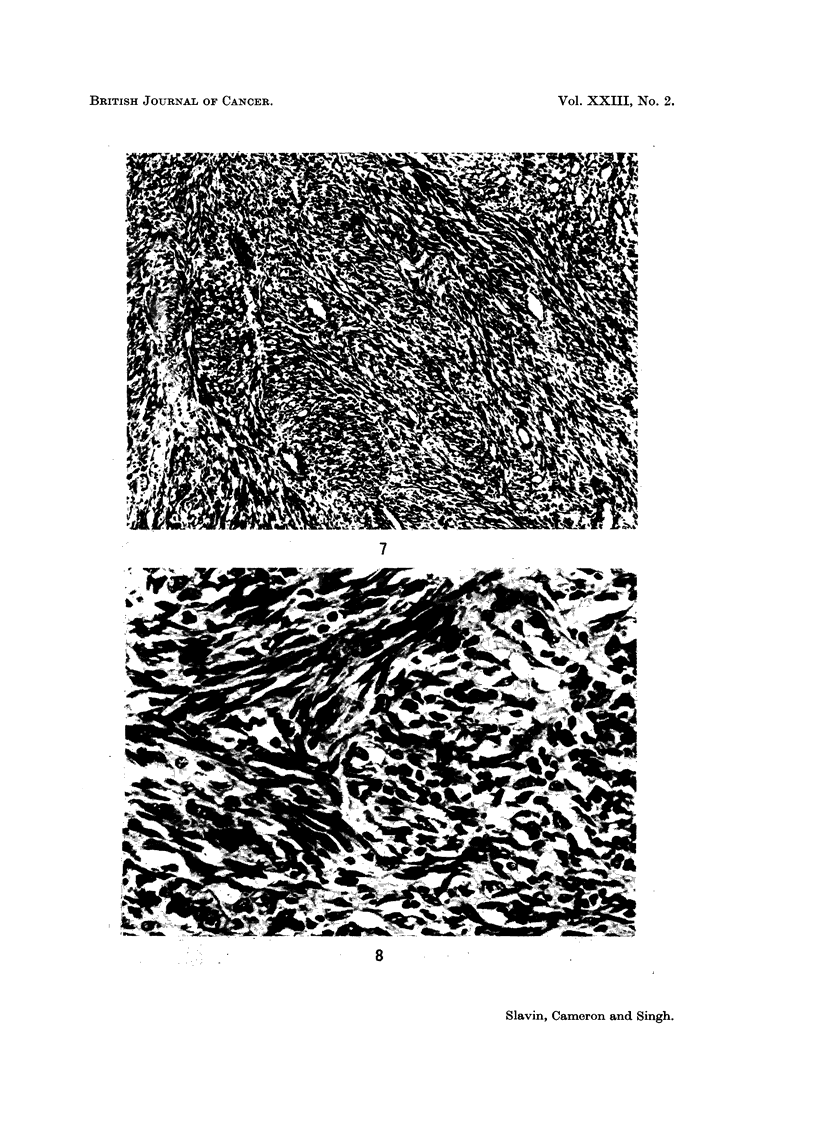

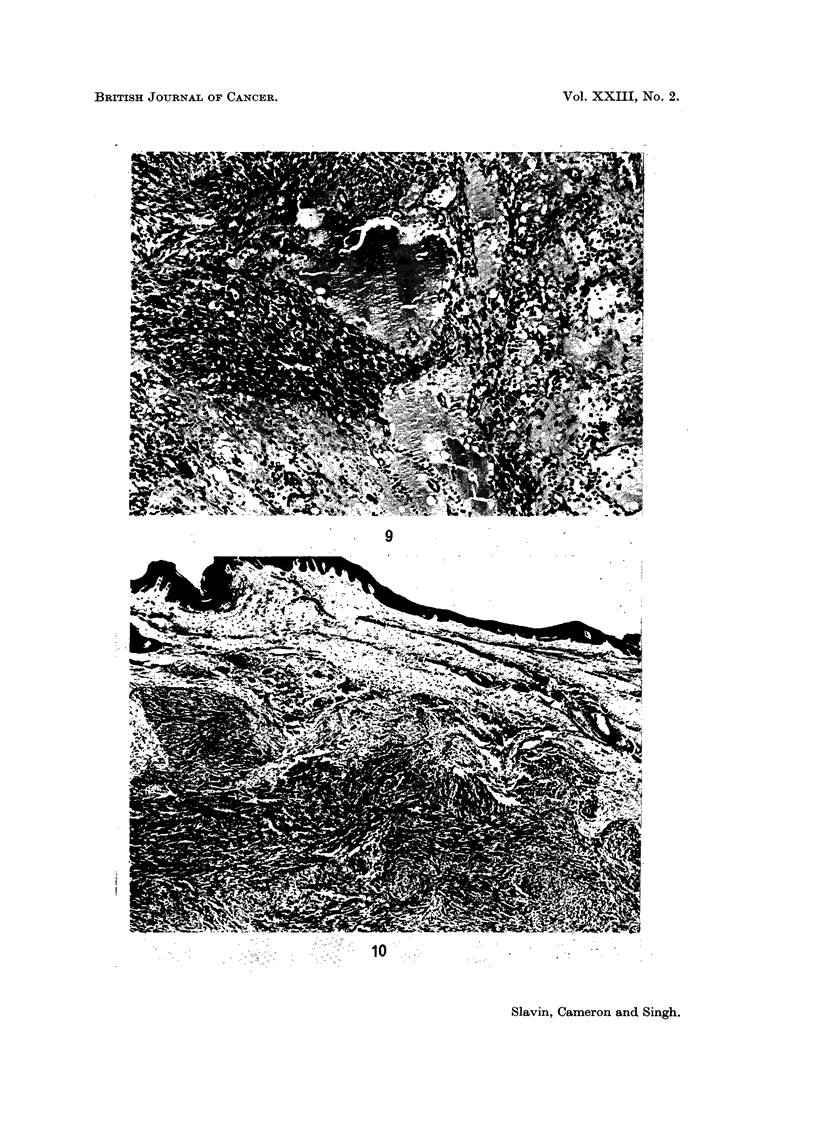

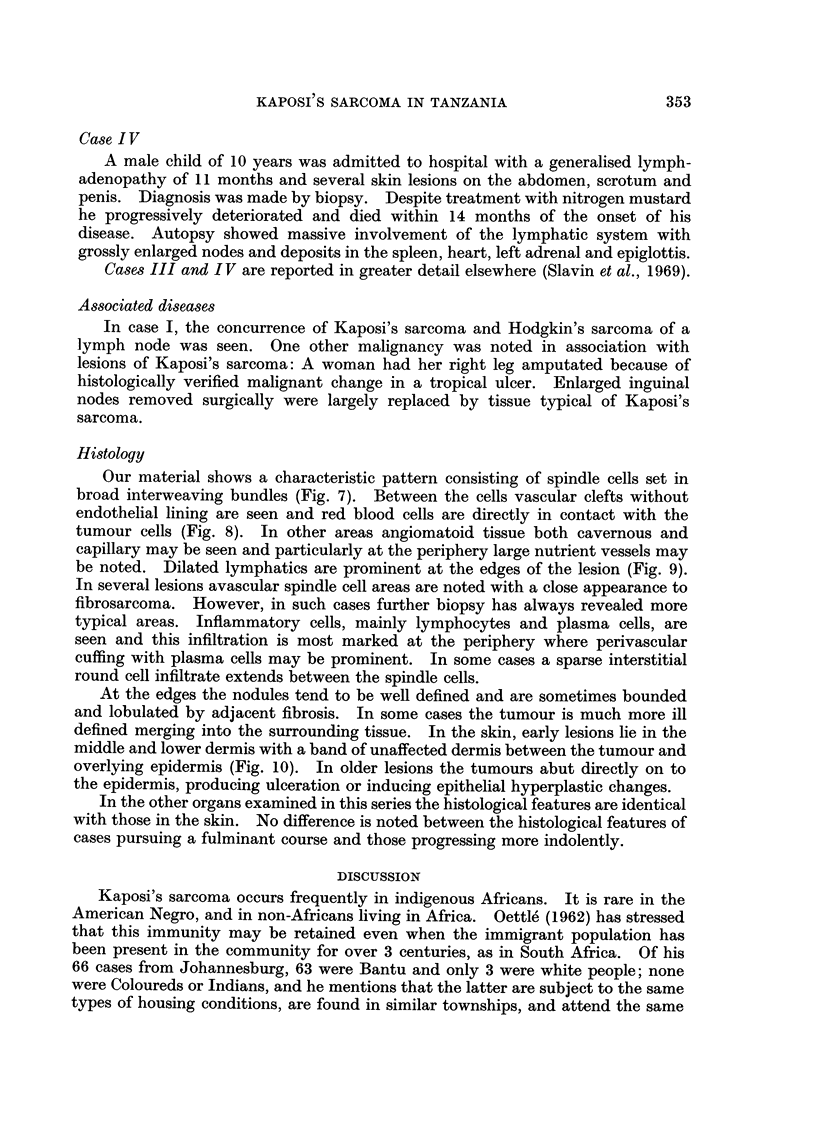

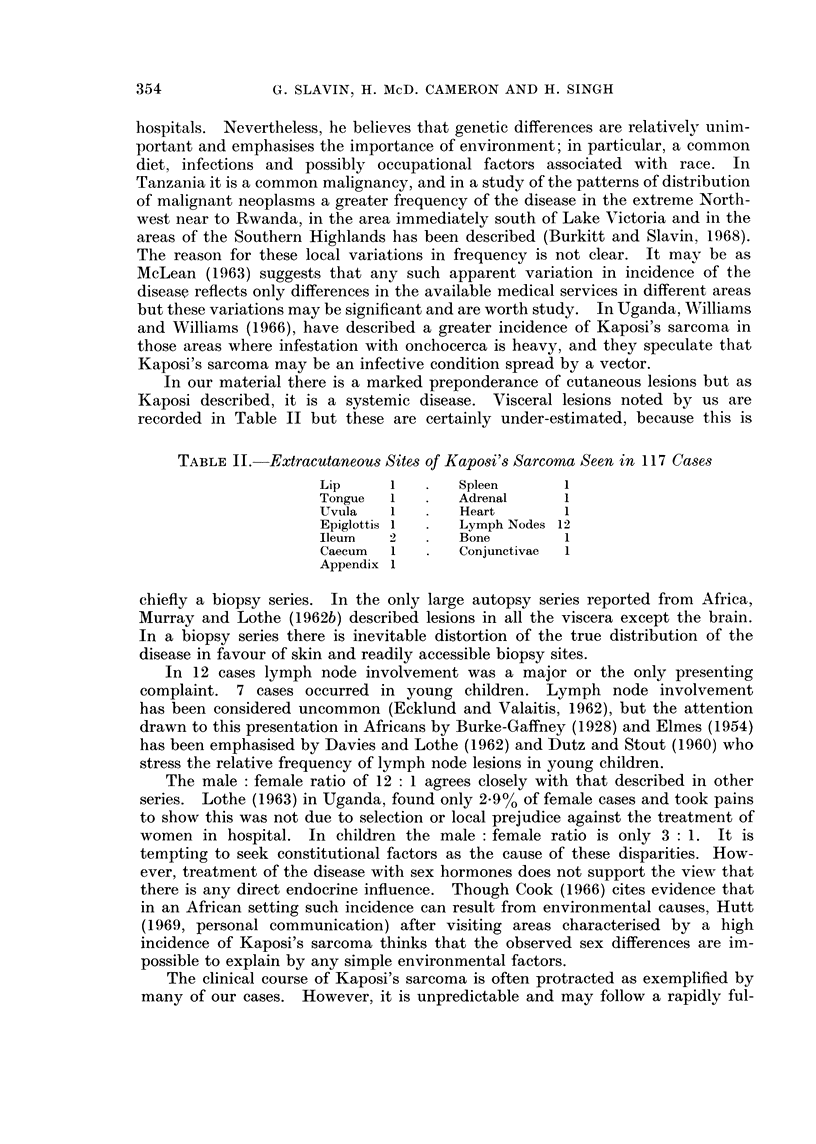

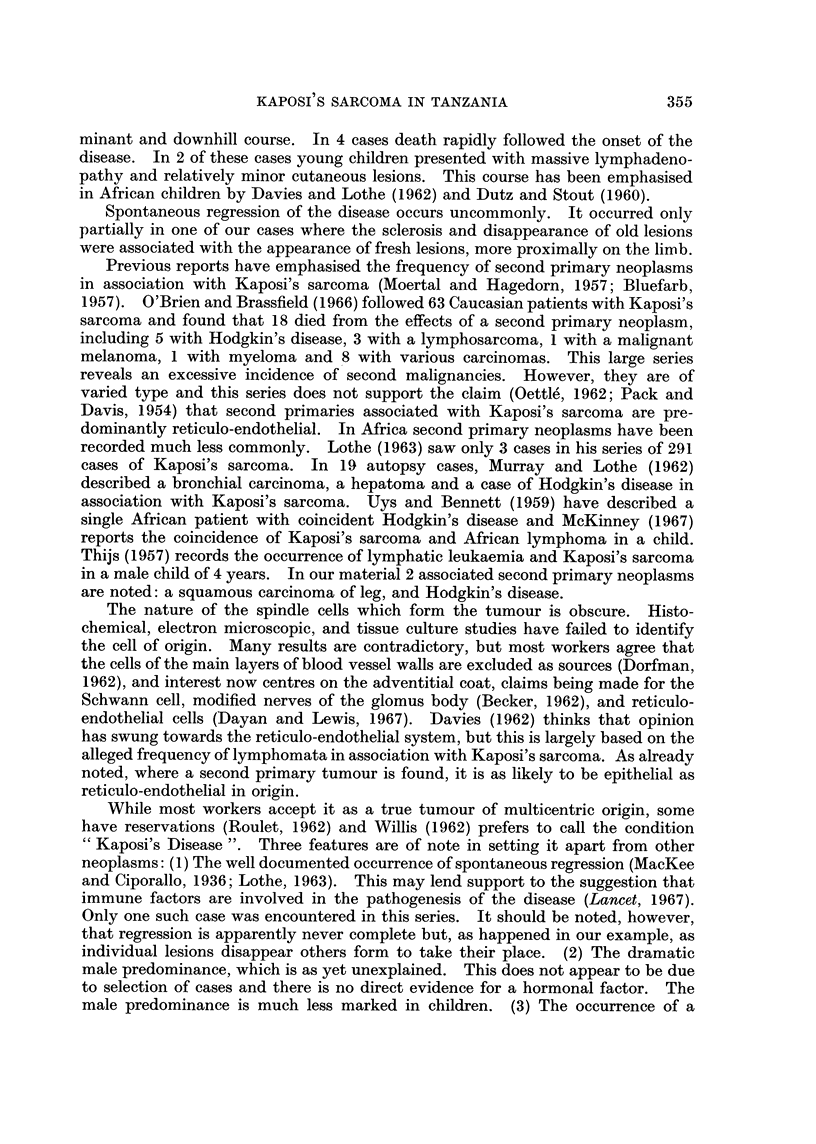

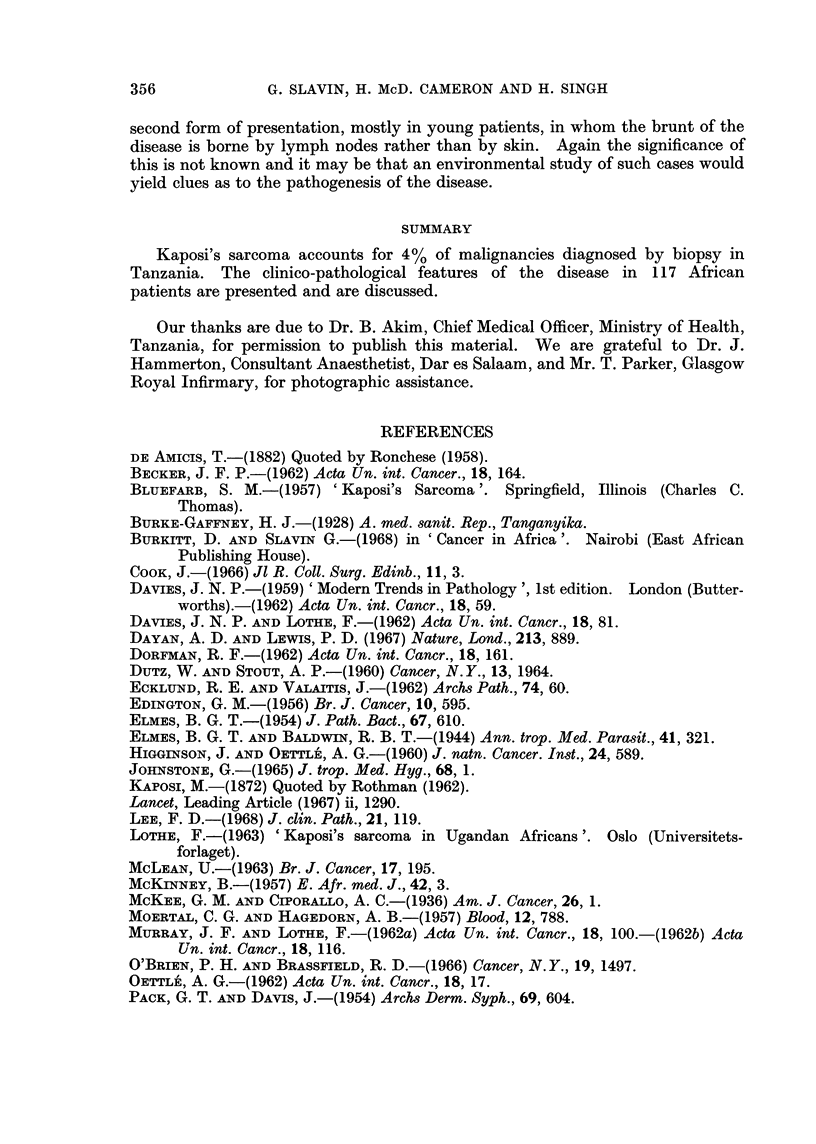

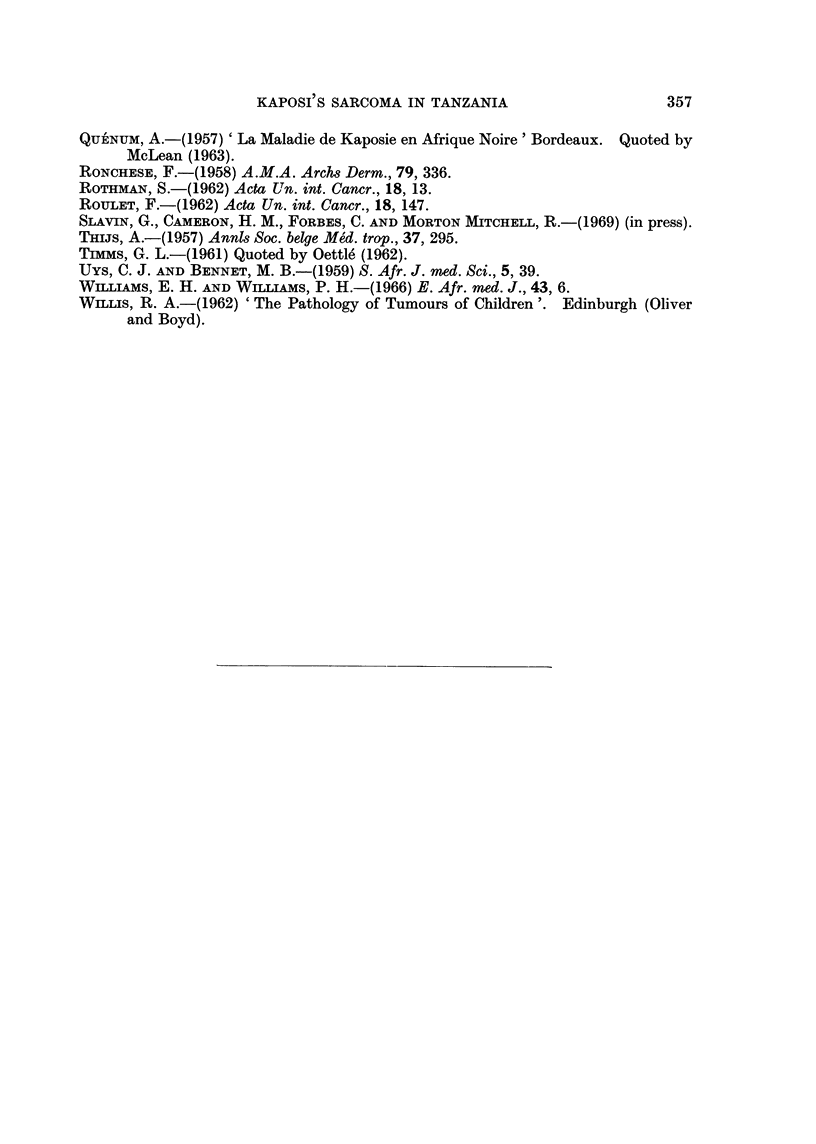


## References

[OCR_00443] Dayan A. D., Lewis P. D. (1967). Origin of Kaposi's sarcoma from the reticulo-endothelial system.. Nature.

[OCR_00450] ELMES B. G. (1954). Kaposi's sarcoma of lymph nodes; a report of two cases.. J Pathol Bacteriol.

[OCR_00454] HIGGINSON J., OETTLE A. G. (1960). Cancer incidence in the Bantu and "Cape Colored" races of South Africa: report of a cancer survey in the Transvaal (1953-55).. J Natl Cancer Inst.

[OCR_00471] MOERTEL C. G., HAGEDORN A. B. (1957). Leukemia or lymphoma and coexistent primary malignant lesions: a review of the literature and a study of 120 cases.. Blood.

[OCR_00478] PACK G. T., DAVIS J. (1954). Concomitant occurrence of Kaposi's sarcoma and lymphoblastoma.. AMA Arch Derm Syphilol.

